# Supplementary material for: Complexome profiling of the Chlamydomonas psb28 mutant reveals TEF5 as an early PSII assembly factor
Source: Plant Cell. 2025 Mar 17;37(6):koaf055. doi: 10.1093/plcell/koaf055 (PMC12164590; doi:10.1093/plcell/koaf055)
Supplement: koaf055_Supplementary_Data [file koaf055_supplementary_data.zip › psb28_interactive.zip!/plots/intensity/qualityPlots/peptides/_Cre06.g254500.t1.1.html]

### Cre06.g254500.t1.1

Trivial name:   
SeqCoverage [median]: 3.80  
Mass: 87.23 kDa  
  
 

| Sequence | MissedCleavage | Mass [Da] | wtIntensity | muIntensity | Score |
| --- | --- | --- | --- | --- | --- |
| ARETPAPAPAPAPAAMR | 1 | 1673.87 | 0.00 | 482.62 | 64.56 |
| LVVSEFEAR | 0 | 1048.56 | 951.40 | 2214.80 | 60.59 |
| SGTAAAAPR | 0 | 800.41 | 215.77 | 0.00 | 65.25 |
|  |  |  | 1167.2 | 2697.4 |  |
